# Tissue distribution of naringenin conjugated metabolites following repeated dosing of naringin to rats

**DOI:** 10.7603/s40681-014-0016-z

**Published:** 2014-08-13

**Authors:** Shiuan-Pey Lin, Yu-Chi Hou, Shang-Yuan Tsai, Meng-Ju Wang, Pei-Dawn Lee Chao

**Affiliations:** 1 School of Pharmacy, China Medical University, No.91 Hsueh-Shih Road, Taichung, Taiwan; 2 Department of Medical Research, China Medical University Hospital, Taichung, Taiwan; 3 Department of Pharmacy, China Medical University hospital, Taichung, Taiwan; 4 Institute of Chinese Pharmaceutical Sciences, China Medical University, Taichung, Taiwan

**Keywords:** Naringin, Naringenin, Sulfates, Glucuronides, Tissue distribution

## Abstract

Background: Naringin is a major antioxidant in *Citrus* fruits and herbs. To clarify molecular forms distributed to various tissues, we investigated tissue distribution of naringin and relevant metabolites in rats after repeated dosing.

Methods: Male Sprague-Dawley rats were orally administered naringin (210 mg/kg) twice daily for eight days. At 6 h post the 17^th^ dose, various tissues including liver, kidney, heart, spleen and brain were collected and analyzed by HPLC method before and after hydrolysis with β-glucuronidase and sulfatase, individually.

Results: The free forms of naringin and naringenin were not detected in all the tissues assayed. Liver contained the highest concentration of naringenin sulfates, followed by spleen, heart, brain and kidney. Naringenin glucuronides were present in liver and kidney, but not in spleen, brain and heart.

Conclusion: The bioavailability of naringenin glucuronides and sulfates supported its application for personalized medicine.

## 1. Introduction

**Fig. 1:**
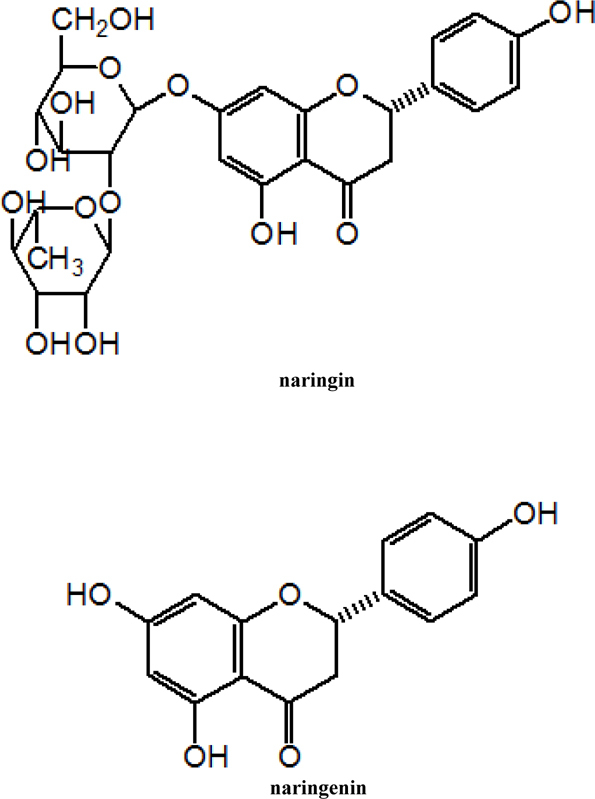
Chemical structures of naringin and naringenin.

Flavonoids, major class of antioxidants, prove interesting to pharmacologists due to various beneficial bioactivities [1,2]. They also draw attention from pharmaceutical researchers via capacity for modulating P-glycoprotein (P-gp) and CYP 3A4 [3-6], both prominent in pharmacokinetics [7, 8]. Naringin (4’, 5, 7-trihydroxyflavanone 7-rhamnoglucoside) is one major flavonoid distributed in *Citrus* fruits like grapefruit (*C. paradisi*) or pomelo (*C. grandis*) and in Chinese herbs like *C. aurantium* and *C. maxima* [9]. Numerous *in vitro* studies report naringin and naringenin, the aglycon of naringin, (structures in Fig. 1) exhibiting benefits: e.g., anticancer [10,11], superoxide scavenging, antioxidation [12,13], antimicrobial effects [14]. Naringin-containing nutraceuticals are increasingly used as dietary supplements, yet based on our prior studies reporting pharmacokinetics of naringin in rabbits and rats, n aringenin sulfates and glucuronides appeared predominately in the blood-stream, with no trace of naringin or naringenin detected [15,16]. Whether bioactivities of naringin and naringenin cited by earlier *in vitro* study can be extrapolated to *in vivo* effects remained unanswered [17]. Regarding major forms in various organs, previous studies report tissue distribution of naringin or naringenin in rats [18-20], yet these followed single dose via intravenous or oral route, findings less than consistent. We analyzed distribution of naringin and relevant metabolites in rat tissue after repeated dosing.

## 2. Materials and methods

### 2.1. Chemicals

Naringin (purity 95%), (±)-naringenin (purity 95%), β-glucuronidase (Type B-1, 666,400 units/g, from bovine liver), sulfatase (Type H-1, 20,000 units/g, from *Helix pomotia*, containing 498,800 units/g of β-glucuronidase) and vanillin were purchased from Sigma Chemical Co. (St. Louis, MO); 5,7-Dimethoxycoumarin (99%) from Aldrich (Milwaukee, WI); acetonitrile, methanol and ethyl acetate of HPLC grade from Mallinckrodt Baker, Inc. (Phillipsburg, NJ); L(+)-Ascorbic acid from RdH Laborchemikalien GmbH & Co. KG (Seelze, Germany). Other reagents were of analytical grade, Milli-Q plus water (Millipore, Bedford, MA) used throughout.

### 2.2. Instrumentation and HPLC conditions

The apparatus was equipped with a pump (LC-10AT, Shimadzu, Japan), UV detector (SPD-10A, Shimadzu, Japan), automatic injector (SIL-10A, Shimadzu, Japan) and Cosmosil C18 column (5 μm, 150×4.6mm, Waters, MA). Detection wavelength was set at 288 nm. Mobile phase consisted of acetonitrile: 0.1% phosphoric acid (36:64, v/v), flow rate 1.0 mL/min for naringenin assay in serum and tissue homogenate.

### 2.3. Drug administration and collection of blood and organs

Six male Sprague-Dawley rats weighing 300-350 g were purchased from the National Science Council, Taipei, and maintained in the China Medical University Animal Center. Naringin was dispersed in warm water for oral administration (210 mg/kg twice daily) for 17 doses via gastric gavage. Finally, rats were fasted overnight before 17^th^ dose, blood and various organs were collected at 6 h after dosing, based on peak time of naringenin conjugates observed by our previous pharmacokinetic study [16]. Immediately after blood collection, systemic perfusion was conducted by pumping normal saline to wash out blood. The organs including liver, kidney, spleen, heart and brain were dissected, blotted dry, accurately weighed, and frozen at -80℃. Animal study adhered to *Guidebook for the Care and Use of Laboratory Animals* (2002) published by The Chinese Society for the Laboratory Animal Science, Taiwan.

### 2.4. Preparation and quantitation of tissue samples

All tissue samples were lyophilized, then chopped into small pieces and milled with normal saline (300 μL/g tissue) by Potter-Elvehjem tissue grinder (Kontes Glass Co.; Vineland, NJ). Homogenates (0.5 mL) were deproteinized with 3-fold methanol. After centrifuge, supernatant was evaporated to dryness under vacuum, then dissolved with 0.5 mL of pH 5 acetate buffer to afford tissue extract. To quantify naringin, tissue extract was analyzed before and after hydrolysis with β-glucuronidase and sulfatase, individually. To quantify naringin conjugated metabolites, tissue extract (100 μL) was mixed with 100 μL of glucuronidase (1000 units/mL in pH 5 buffer) and/or sulfatase (containing 1000 units/mL of sulfatase and 24,940 units/mL of glucuronidase in pH 5 buffer), 100 μL of ascorbic acid (150 mg/mL) and incubated at 37°C for 2 h, mixture added with 900 μL of acetonitrile (containing 6 μg/mL of vanillin). For free form determination of naringin, homogenate was treated with pH 5 acetate buffer without incubation with glucuronidase or sulfatase and processed as per procedure detailed above. Acetonitrile layer evaporated under N_2_ gas to dryness was reconstituted with appropriate volume of acetonitrile, 20 μL subjected to HPLC analysis. To quantify free form naringenin, tissue extract was determined before hydrolysis with sulfatase or β-glucuronidase. Briefly, 100 μL of the deproteinized tissue extract was acidified with 100 μL of 0.1 N HCl and partitioned with 500 μL of ethyl acetate (containing 2.0 μg/mL of 5,7-dimethoxycoumarin as the internal standard). The ethyl acetate layer was evaporated under N2 gas to dryness and 20 μL was subject to HPLC analysis.

To quantify naringenin glucuronides, 100 μL of buffer containing tissue extract was mixed with 100 μL of β-glucuronidase (1000 units/mL in pH 5 buffer), 100 μL of ascorbic acid (150 mg/mL) and incubated at 37°C for 2 h. To quantify naringenin sulfates/glucuronides, 100 μL of the buffer containing tissue extract were mixed with 100 μL of sulfatase (containing 1000 units/mL of sulfatase and 24,940 units/mL of glucuronidase in pH 5 buffer), 100 μL of ascorbic acid (150 mg/mL), and incubated at 37°C for 1 h. After hydrolysis, procedure was the same as described above for free form naringenin. For calibrator preparation, 100 μL of tissue standards with various concentrations of naringenin were spiked with 100 μL of pH 5 acetate buffer, 100 μL of ascorbic acid (150 mg/mL), then added to 100 μL of 0.1 N HCl. Later procedure followed that described above. Calibration graph was plotted by linear regression of peak area ratios (naringenin to internal standard) against concentrations of naringenin.

**Table 1:** Concentrations (nmol/mL for serum and nmol/g for tissues) of naringenin glucuronides (G) and naringenin sulfates (S) in serum and various organs at 6 h following 17^th^ dose of naringin (210 mg/kg) in six rats.

Metabolites		serum	liver	spleen	heart	brain	kidney
G	Mean	3.7	2.6	0	0	0	0.9
	S.E.	0.1	0.1	0	0	0	0.1
S	Mean	0.7	8.2	3.4	1.4	1.1	0.1
	S.E.	0.0_4_	1.5	0.7	0.2	0.3	0.0_4_

### 2.5. Data analysis

Concentrations of naringenin glucuronides and naringenin sulfates in each tissue were expressed in μmol per gram of tissues (μmol/g). Ratios (mL/g) of concentrations of naringin glucuronides and naringin sulfates in various organs (μmol/g) to those in serum (μmol/mL) were calculated, although units differed.

## 3. Results

Quantitation method of naringenin in each tissue was established and optimized. Good linearity was obtained at concentration 0.4-50.0 μg/mL of naringenin in each tissue. Validation of assay indicated all coefficients of variation (CVs) and relative errors of intra-run and inter-run analysis below 2.2% and 18.1%, respectively. The LLOQ and LOD of naringenin were 0.40 and 0.02 μg/mL, respectively, in all tissues. Results indicated neither naringin nor naringenin in liver, spleen, heart, brain and kidney, molecular forms in tissues as naringenin sulfates and naringenin glucuronides. Table 1 plots concentrations of naringenin sulfate and naringenin glucuronide in serum and various organs 6 h post 17^th^ dose. Results indicate the liver with highest ratio of naringenin sulfates, followed by spleen, heart brain, and kidney. Naringenin glucuronides were present in liver and kidney, not in spleen, brain, or heart.

**Fig. 2:**
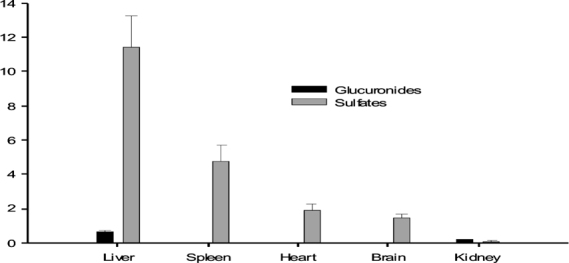
Relative mean (±S.E.) ratios of concentrations of naringenin glucuronides and naringenin sulfates in various tissues to serum at 6 h after repeated doses of naringin (210 mg/kg) twice daily for nine days to six rats.

Regarding analysis of serum collected 6 h post 17^th^ dose, quantiation showed naringenin glucuronides and naringenin sulfates as major forms, whereas naringin and naringenin were not present, which was consistent with previous studies [15, 16]. Fig. 2 shows ratios of naringenin glucuronides and naringenin sulfates in each tissue to those in serum. Among assayed organs, liver had the highest naringenin sulfate level, about 10.7-fold; spleen, heart and brain had higher concentrations of naringin sulfates than serum by 386, 100, and 57 %, respectively. In regard to distribution of naringenin glucuronides, conversely, liver and kidney contained lower concentrations than serum by 30 and 76%, respectively.

## 4. Discussion

Quantitation method of naringenin glucuronides and sulfates in various tissues was established and validated. Due to considerable amount of β-glucuronidase in sulfatase (Type H-1), this enzyme hydrolyzed both sulfates and glucuronides simultaneously. Results of both hydrolysis with sulfatase and glucuronidase could yield individual naringenin sulfate and naringenin glucuronide concentrations in each tissue specimen. Quantitation of serum and collected tissues showed that after repeated dosing, free forms of naringin and naringenin were not detected in both bloodstream and all assayed organs; major molecular forms were naringenin sulfates and naringenin glucuronides. In serum, naringenin glucuronides were predominant; in liver, spleen, heart and brain, narigenin sulfate was the major form. Findings imply naringenin glucuronides deglucuronidated and then sulfated in these organs.

Among assayed organs, the liver contained higher naringenin sulfate and naringenin glucuronide concentrations than other organs, which concurred with previous studies [19] and indicated these naringenin conjugates have higher protein binding with liver proteins. In the liver, concentration of naringenin sulfates was higher than that of naringenin glucuronides by 240%. In spleen, heart, and brain, naringenin glucuronides were not detected, meaning naringenin released through hydrolysis with sulfatase in these organs was solely from naringenin sulfates. It can thus be assumed that when naringenin glucuronides entered liver, spleen, heart, or brain from circulation, they were hydrolyzed by glucuronidase, then sulfated by sulfotransferase in these organs [21,22]. In sum, liver, spleen, heart and brain contained narigenin sulfates as principal metabolites of naringin. Therefore, bioactivities of naringenin sulfates in liver, spleen, heart and brain warrant more investigations. In the kidney, naringenin glucuronides and naringenin sulfates manifested far lower concentrations than those in serum, indicating that only a little fraction of glucuronides or sulfates had entered the kidney. This finding was not consistent with previous study reporting moderate concentrations of naringenin glucuronides detected in kidney, liver and brain at 2 h post oral dose of naringenin in rats [18]; future studies must clarify.

Our study revealing absence of naringenin in all assayed tissues was not in good agreement with prior research detecting naringenin in tissues after single dose of naringin [19]. This discrepancy might arise from variant dosage or detection method: i.e., 17 doses of naringin might modulate expression of UDP-glucuronosyltransferase or sulfotransferase and result in more extensive metabolism [23]. Repeated dosing of naringin to rats yielded wide distribution of naringenin sulfates to various organs, while naringin and naringenin reached no organs. We identified chemical nature and concentration of naringenin conjugates in tissue; these may disclose pharmacological roles of putative active metabolites after chronic dosing of naringin.

### Acknowledgements

The work was in part supported by National Science Council (NSC 102-2320-B-039-014-MY2, NSC 102-2320- B-039-008), and China Medical University, Taiwan, R.O.C. (CMU101- N2-08, CMU102-S-16).

**Declaration of Interest:** Authors declare no conflicts of interest for this work.
